# Pennsylvania State Veterinary Medical Association

**Published:** 1900-12

**Authors:** 


### SOCIETY PROCEEDINGS.

### PENNSYLVANIA STATE VETERINARY MEDICAL ASSOCIATION.

(Continued from page 709.)

*Reports of Committees* (continued).

*Sanitary Science and Police.* The importance of and progress in this line of work was read as a report from Dr. Jacob Helmer, as follows: My report, also two communications from members of the Committee on Sanitary Science and Police, one received from Dr. J. L. Bradley, of Mercersburg, Franklin County, the other from Dr. H. P. Keely, of Schwenksville, Montgomery County, have been forwarded to the Association through our worthy Secretary, Dr. Ranck.

Dr. L. A. Klein, of Philadelphia, replying to my request for a report, promised to send one, but at this writing no further communication has been received. While we should have been happy to have received the doctor's paper, we hope that he has decided to attend the meeting and present the report in person. It is also hoped that communications from other members of our committee who have not reported to me may be personally presented for the pleasure and the instruction of the Association. Could each member of the committee have sent a report or communication it would have been my pleasure as chairman to weave the material into one paper, but since some may present their reports in person or through other members I deemed it best to have each communication read by itself.

In Lackawanna County (the one in which I practice) there has not been anything unusual in the country sections this season, unless it is influenza. In my immediate neighborhood—Scranton and suburbs—there has been an unusual amount of this disease, which assumed the special form of pink-eye. The enzoötic this year is very mild. While we usually have the disease present with us during the spring and fall, this year it has flourished during the hot months of summer. It is interesting to report that in scores of cases treated not a single complicated one has come under my observation. A farmer reported at my office about two months ago that some of his cattle were suffering with what appeared to be foot-and-mouth disease. From his description it resembles very closely the description given us of the European plague. He reported that his herd was convalescent, members of it having been afflicted with it from one to two weeks. The trouble was benign, and tended to recover without treatment. I did not meet the man personally. He did not leave his address, and I heard nothing further of the malady.

But while the livestock interests throughout Lackawanna County have not suffered in an unusual degree this year from any fatal maladies, the neighboring county, Wayne, is passing through an uncommon experience with what appears to be anthrax. Perhaps there is no county in the State which can be proud of as many beautiful lakes as Wayne. Over five hundred fine bodies of water there have been graphically described. In this undulating country miniature swamps and marshes exist abundantly in the cultivated districts. The marshes are the bottoms of beautiful lakes of former years. The sloping land also terminates in ravines and swales. Little marshes may be seen leading into these from the hillsides. Generally speaking, these sections have an impervious subsoil. The ground is rich and bears coarse grasses of edible varieties in abundance. In a dry season the vegetation here would flourish, since there is sufficient water and moisture in the soil to supply the roots of the grasses. Cattle would crave the lowland in preference to the upland pastures, because a more succulent and tender morsel could be found there. The summer in Wayne County has been very dry, and the herbage on the upland has become tough and innutritious, and the cattle naturally tend to rummage in the marshes. In a section of country where the conditions for anthrax exist in the surroundings and soil, and when supplemented by drought, may not other varieties of vegetable parasites and miasmatic poisons, of whatever nature they be, find opportunity to exist in unusual abundance upon the grasses? It is possible, also, that a poisonous weed or shrub may grow in the bottoms to which the cattle have been led this season. Something poisoned many a herd of cattle in Wayne County, and the question is, Was it anthrax or some other malady? In order that you may understand the subject more in detail I will give you the substance of my first report to Dr. Pearson of the investigation of the disease on the farm of Mr. J. R. Mills, in Oregon township, Wayne County, Pa., on the 24th instant:

"I found that the disease which afflicts Mr. Mills' herd is confined to a single field or pasture containing about twenty acres. The field has been used for pasturage at least five years, and until this season no fatal sickness had appeared there. In a herd of eighteen head, composed of grade and common stock, quite uniformly one and two years of age, there have been seven deaths up to the time of my visit to the farm. The field in which the disease appeared is approximately in the form of a rectangle. It slopes, and the whole lower part forms one side of a swale. The field is three-quarters dry. The remaining fourth is marshy ground, which extends upward from the swale nearly to the upper border of the field. The marsh does not occupy the centre, but rather one side—the right side of the field as viewed from the swale. The earth in the marsh is dark in color and is soft. The feet of the cattle sink into it. There are many little bogs with holes between them, containing water. The vegetation in the marsh is thick and rich. It consists mainly of coarse, wild grasses, interspersed with some tame varieties. The marsh affords the best fodder in the field, especially during the drought which the locality is now experiencing. The rest of the field is rough and rocky. There is an abundance of croppage, but it is dry and tough as compared with the grass in the marsh. A very careful search did not reveal any poisonous shrubs that had been eaten by the cattle. There were some laurels, but the leaves were intact. The season for this section of Wayne County has been unusually dry. All summer there has been no rain sufficiently to soften the ground on Mr. Mills' farm. The rainfall has consisted of a series of showers, some hard and some light. These showers were almost invariably followed by warm sunshine, which soon dissipated the good effects of the rain. The weather on cloudy days has been hot and muggy. But while three-fourths of the pasture field in question was hard and dry, the remaining one-fourth—the marsh—while more dry and exhausted than ever before, still contained considerable water and moisture. The stock in this field, while not exactly plump and round, were in very good condition, considering the season. It would appear that the bulk of the nourishment was furnished by the marsh. In addition, the cattle looked healthy, with the exception of one yearling, which appeared a little thin and was quite dull and dumpish. This animal appeared to be a fresh victim of the malady. Mr. Mills has been in the habit of separating the sick from the healthy animals as soon as a case presented itself. The sick are enclosed in a pen in a place where they cannot possibly contaminate other cattle. Of this method I approve most heartily. Case No. 1 was found dead in the field about two weeks ago. It had evidently died a day or two previously. There were no external swellings, but the body was bloated and the legs puffed. There was an exudate of bloody material from the nostrils. The last passage of fecal matter resembled tar, as if it had been mixed with black blood. Case No. 2 was found dead a day or two later. The appearances were the same. Case No. 3 was also found dead. Bloody discharges the same; body and limbs bloated. Skin of a dark-green hue. These three animals died during the first week. During the second week four became ill and died. These were observed while alive. The deaths followed each other from day to day, with evidently an increase in the percentage of death-rate. The symptoms showed by these four young animals during their short period of illness were very uniform. In no instance did a case live over five days. Death occurred in from three to five days.

"The symptoms as observed and told me by Mr. Mills are as follows: Dulness was about the first thing to indicate anything wrong. The eyes had a glassy appearance. The muzzle was hot and dry. The back along the spine felt particularly warm. No chills were observed, but the coat became dry and staring. In one case a swelling about the size of the hand appeared on the left side of the body just back of the elbow. Dark-colored blood oozed from the swelling. Mr. Mills did not notice whether this swelling was crepitant. In other cases the ankle was swollen, but external swellings were the exception in all these cases, and principally affected the ankles. In no case did the skin around the swollen ankle crepitate on pressure. The appetite was very feeble; only a little meal or apple was partaken of. Water was only sparingly consumed. Grating or grinding of the teeth was almost a constant symptom. As the disease advanced the animals were listless. There was no pain; the back was arched. Emaciation, exhaustion, and weakness followed rapidly. In walking the animals had a staggering gait. The force of the disease seemed to be exerted upon the hind parts. There was no cough, no difficult breathing. Finally, a bloody discharge issued from one or both nostrils, and the passage resembled tar mixed with blood. The urine was voided frequently and was bloody in appearance, but not particularly of a dark color. Shortly after the appearance of these latter symptoms death ended the scene.

"Case No. 7 died during the night previous to the day of my arrival. It was buried in the morning. We had it exhumed and held an autopsy. Externally there were no swellings. The carcass appeared quite normal. On opening the abdominal cavity, serum, colored with blood, flowed freely. The subserous connective tissue in the neighborhood of the kidneys was infiltrated with blood and serum. Bloody extravasations existed in the mesentery and upon the outside of the duodenum, colon, and rectum. A

few of the mesenteric glands were enlarged; they were engorged with black blood. Dark, hemorrhagic spots were found on the muscles under the spine and on the muscles lining the cavity of the pelvis. The small intestines, with the exception of the duodenum, were quite free from alterations. Portions of the rumen as well as of the large intestines were black and blue, and were moist, with a dark-colored exudate on the inner walls. The kidneys seemed slightly larger than normal, but on section looked natural. There was no engorgement, and the fluid issuing from the cut surface was of a bright-red color. It seemed like urine thinned with blood. The liver was enlarged, very friable, in places mushy. Its color resembled pale clay; not black or dark. The spleen was normal in size, and with a section of the kidney, liver, and ear it has been shipped to you intact. The lymphatic vessels of the portal system were distended, and spots of the retroperitoneal tissue were dropsical. The peritoneum was inflamed in patches and the subperitoneal tissue was infiltrated with serum and blood. The organs of the thoracic cavity, aside from numerous pin-points of congestion in the thoracic cavity, were red, as were also the capillary vessels of the peritoneum of the small intestines. The brain, larynx, and trachea were not examined. The body as a whole was not decomposing rapidly; in fact, it appeared quite fresh.

"Physical examination of Case No. 8 revealed dulness, eyes glassy, and diminished appetite. The conjunctiva was of a yellowish-brown color, and here and there could be observed a petechial point. Non-crepitant swelling, uniformly distributed around the right hind ankle, was observed. The temperature was 105° F. and the pulse frequent and sthenic. This case was ordered to the pen and placed under treatment.

"Since a positive diagnosis of anthrax in many of its forms depends upon the finding of the specific germ, I did not make a positive statement, reserving such a positive decision until after we shall have heard from the bacteriological examination of the specimens sent you. Of the infectious character of the disease there seems no doubt. The objective symptoms, as well as the results of the autopsy, point mainly to death caused, in this case, by subacute bacteridian anthrax. It is evident that the malady had its origin in the field previously described. To stop the invasion, therefore, the first thing ordered done was to remove the stock from this into another field. A fence is to be built around the dead carcasses that were buried near each other, just across the fence in the adjoining field, while the grass and ground around them are treated liberally with lime. Those found dead were buried in the field where each victim fell. This pasture is now abandoned.

"In conclusion, allow me to say that the section of Wayne County where these cases occurred seems peculiarly adapted to furnish the conditions necessary for the development of anthrax. The drainage from the hills runs into the swale. The stream in the bottom of the swale, while not exactly stagnant, moves very slowly, or, rather, leaks out. The marshes that extend from the swales upward into the pasture fields are especially dangerous; but, more than this, the fact that the water in the bottom of the swale appears lower this season than usual, thus permitting some patches of ground which were submerged in the early part of the season to become exposed to the air and sunlight, thus giving rise to a rich vegetation upon them, have probably been the sources for the development of the dangerous vegetable parasite, the mischief-maker, which these young cattle have taken into their systems by feeding upon the bogs. Add to this the natural formation of the ground, the peculiar lay of the land, and also the fact of an alternating wet and dry season, especially the latter—an unusual season for this section of Wayne County—and we have some good reasons to contemplate why cattle should die of anthrax here as well as in other portions of the State of Pennsylvania. Kindly let me hear from you as soon as the bacteriological investigation has been completed. If anthrax is decided upon, complete instructions must be given Mr. Mills as early as possible."

Dr. Pearson subsequently reported that the specimens had spoiled in transit, and requested me to send fresh ones. He also took the position that the disease might not be anthrax, inasmuch as it resembled a few similar outbreaks in the State that were now known not to be infectious. Vaccine was, however, promptly forwarded to prevent further mischief by the disease in case it should be anthrax. Two head from the remaining part of this herd have been taken sick since being removed to upland ground. The last case was taken seventeen days after the change. We are on the lookout for microscopical material, and do not wish to see the season pass without the problem being solved. Finally, speaking for myself, I believe the disease is anthrax, since not one of the elements necessary to show a case of anthrax is missing, unless the specific germ cannot be demonstrated by microscopical examination, or another germ is found in its place that causes a similar disease.

From Dr. John L. Bradley, of Mercersburg, Franklin County: I would report to you that I am in the midst of an outbreak of rabies, with about fifteen bitten dogs and a lot of contrary people to deal with; one man and a child bitten. So far nobody has been sent to New York for inoculation. Tuberculosis exists to some extent, but a great many cases have been put out of the way by the State Board. By the way, I think this Board should be commended for the good work it has done, also its most efficient head, Dr. Leonard Pearson. Cows for dairy purposes are coming in from Maryland and other States, and I have some work in that line, also some requests from private families asking that their animals be subjected to the tuberculin-test before using the milk further. An outbreak of some disease among swine, which the State Board is now investigating, and report that they find nothing on post-mortem; also on two farms an outbreak of specific ophthalmia in the cattle, all of which were blind for a time, but are now improving. I believe that is all I have from a sanitary science point of view.

From Dr. H. P. Keely, of Schwenksville, Montgomery County: Our county is blessed with freedom from many of the contagious diseases of cattle except tuberculosis, of which we have our full share, although it appears to be growing less, and I think that most of the worst stables have been cleaned out in this section. We had considerable of that peculiar form of influenza among shipped horses this spring, with a pretty high rate of mortality. We have had about our usual share of parturient paralysis, but with the use of the potassium iodide treatment there have been more recoveries, and I believe that with the prophylactic treatment (lighter feeding during the dry period and physic shortly before calving), which we advise our clients to use, the number of cases of milk-fever are decreasing.

Dr. C. C. McLean made a short verbal report. These reports were discussed by Drs. Jobson, Pearson, and Hoskins.

(To be continued.)

